# Pharmacokinetics (PK), Pharmacodynamics (PD) and Integrated PK/PD Modeling of a Novel Long Acting FGF21 Clinical Candidate PF-05231023 in Diet-Induced Obese and Leptin-Deficient Obese Mice

**DOI:** 10.1371/journal.pone.0119104

**Published:** 2015-03-19

**Authors:** Yan Weng, Jeffrey R. Chabot, Barbara Bernardo, Qingyun Yan, Yimin Zhu, Martin B. Brenner, Chandra Vage, Alison Logan, Roberto Calle, Saswata Talukdar

**Affiliations:** 1 Cardiovascular Metabolic and Endocrine Diseases (CVMED) Research Unit, Pfizer Worldwide Research & Development, 610 Main Street, Cambridge, Massachusetts, 02139, United States of America; 2 Pharmacokinetics, Dynamics, and Metabolism, Pfizer Worldwide Research & Development, 610 Main Street, Cambridge, Massachusetts, 02139, United States of America; Johns Hopkins University School of Medicine, UNITED STATES

## Abstract

Pharmacological administration of fibroblast growth factor 21 (FGF21) improves metabolic profile in preclinical species and humans. FGF21 exerts its metabolic effects through formation of beta-klotho (KLB)/FGF receptor 1c FGFR1c complex and subsequent signaling. Data from various *in vitro* systems demonstrate the intact C- and N-terminus of FGF21 is required for binding with KLB, and interaction with FGFR1c, respectively. However the relative roles of the termini for *in vivo* pharmacological effects are unclear. Here we report PF-05231023, a long-acting FGF21 analogue which is unique in that the half-life and subcutaneous (SC) bioavailability of the intact C-terminus are significantly different from those of the intact N-terminus (2 vs. 22 hr for half-life and 4~7 vs. ~50% SC bioavailability). Therefore, this molecule serves as a valuable tool to evaluate the relative roles of intact C-terminus vs. N-terminus in *in vivo* pharmacology studies in preclinical species. We determined the effects of PF-05231023 administration on body weight (BW) loss and glucose reduction during an oral glucose tolerance test (OGTT) following SC and intravenous (IV) administration in diet-induced obese (DIO) and leptin-deficient obese (ob/ob) mice, respectively. Our data show that the intact N-terminus of FGF21 in PF-05231023 appears to be sufficient to drive glucose lowering during OGTT and sustain BW loss in DIOs. Further, PK/PD modeling suggests that while the intact FGF21 C-terminus is not strictly required for glucose lowering during OGTT in ob/ob mice or for BW reduction in DIO mice, the higher potency conferred by intact C-terminus contributes to a rapid initiation of pharmacodynamic effects immediately following dosing. These results provide additional insight into the strategy of developing stabilized versions of FGF21 analogs to harness the full spectrum of its metabolic benefits.

## Introduction

Fibroblast growth factor 21 (FGF21) is a member of the FGF19 subfamily that was discovered to be a critical metabolic regulator for maintenance of glucose and lipid homeostasis [1], thus emerging as a promising novel class of therapeutic for complex metabolic diseases such as type 2 diabetes (T2D) and obesity [2,3]. The beneficial effects of native FGF21 and FGF21 analogues in normalizing glucose and lipid homeostasis have been demonstrated in a variety of preclinical metabolic disease models, including DIO mice, ob/ob mice, db/db mice, diabetic NHP and obese NHP [4–8]. Consistent with the metabolic benefits observed upon pharmacological administration of FGF21 in preclinical species, a recent clinical trial demonstrated robust effects of a stabilized FGF21 analog, LY2405319, in reducing hyperlipidemia and promoting body weight loss in obese T2D subjects [2].

FGF21-mediated biological effects are believed to be mediated through formation of an FGF21/ beta-klotho (KLB)/FGF receptor 1c FGFR1c complex and subsequent signaling [9,5,10]. Native FGF21 is composed of 181 amino acids with a β-trefoil core structure that is conserved in other FGF family proteins and free C- and N-termini that are unique to FGF21 [11,12,10]. The C-terminus of FGF21 is susceptible to proteolytic cleavage and the resultant metabolite is ~200 fold less potent in vitro [13–15]. Moreover, the in vivo half-life of intact native FGF21 is less than 2 hr across multiple species and therefore not ideal for development as a therapeutic for chronic metabolic diseases, such as T2D and obesity. As a result, a number of long-acting FGF21 analogs, including protease stabilized protein [7], Fc-fusion [16], PEG-conjugate [17], and antibodies have been generated and tested in a variety of preclinical species to harness the metabolic benefits of the molecule [18].

PF-05231023 is a long-acting FGF21 analog developed by conjugating two molecules of modified FGF21 [dHis/Ala129Cys] to an antibody scaffold, CovX-2000 [6]. The pharmacokinetics (PK) of PF-05231023 molecule were characterized using an ELISA that measures exposure of drug-related molecules containing the mid-region of FGF21 molecule attached to CovX-2000. Data from ELISA showed PF-05231023 had a prolonged in vivo half-life compared to native FGF21. However, the aforementioned ELISA did not provide information on circulating levels of intact FGF21 C- and N-terminus, believed to be required for molecular interactions with KLB and FGFR1c, respectively. In the current study, two distinct ELISAs were used to measure the exposure levels of intact FGF21 C-terminus (CT) and N-terminus (NT), respectively. The pharmacokinetics of intact CT and NT were assessed in DIO and ob/ob mice following either SC or IV administration. The pharmacological effects of PF-05231023 following SC vs IV administration were also assessed in these two models.

To interpret the pharmacodynamic findings in the context of NT and CT of FGF21, integrated PK/PD models incorporating intact CT and NT PK parameters were developed to allow for estimation of relative contributions of intact FGF21 molecules and potential active metabolites with intact NT alone. Modeling of the apparent potencies of intact FGF21 and NT alone shows that the NT alone is sufficient to drive the observed glucose lowering during OGTT and BW lowering, even with a significant right shift of potency. This is consistent with *in vitro* observations that FGF21 molecules with C terminal truncations (up to 1–171) and intact NT retain the same E_max_ as intact FGF21, but with significant right-shifted potencies [14,15]. These results provide additional insight into the strategy of developing stabilized versions of FGF21 analogs and harnessing the full spectrum of metabolic benefits without eliciting any potential undesired side effects.

## Materials and Methods

### Reagents

PF-05231023 was produced by Pfizer Biotherapeutics Center of Excellence at St. Louis, MO. Mouse anti-CVX-2000 idiotypic antibody, mouse anti-FGF21 C-terminus antibody, and mouse anti-FGF21 N-terminus antibodies were generated by Pfizer.

### Animal studies

All animal studies were conducted in accordance with animal care and use protocols approved by the Institutional Animal Care and Use Committee (IACUC) of Pfizer, Inc. All procedures performed on any animals were in accordance with regulations and established guidelines and were reviewed and approved by a Pfizer Institutional Animal Care and Use Committee and all experiments within this manuscript were undertaken to minimize animal suffering during the experiment.

### Pharmacokinetic studies, assays and analyses

The PK properties of PF-05231023 were evaluated in male 9–10 week old ob/ob and DIO mice following a single IV or SC injection of 3 or 10 mg/kg. Plasma samples were collected predose and 0.25, 1, 2, 6, 24, 48, and 72 hours post-IV dose, or predose and 4, 6, 8, 24, 48 and 72 hours post-SC dose in DIO mice. Plasma samples were collected predose and 0.083, 1, 4, 8, 12 and 24 hours post-IV dose, or predose, 2, 4, 6, 8, 12 and 24 hours post-SC dose in ob/ob mice. All PK samples were stored at -80°C until analysis. Two distinct sandwich ELISAs were used to quantify the intact CT and NT of PF-05231023 in plasma. Briefly, to quantify of plasma levels of PF-05231023 with an intact CT, drug was captured using mouse anti-CovX-2000 idiotypic monoclonal antibody, then detected with a monoclonal anti-FGF21 CT antibody. Similarly, to quantify plasma levels of PF-05231023 with an intact NT, drug was captured using mouse anti-CovX-2000 antibody and detected with a monoclonal anti-FGF21 NT antibody. The quantitation range was 200 to 5000 ng/ml with a lower limit of quantitation (LLOQ) at 200 ng/ml for both assays at MRD of 1:40.

All pharmacokinetic parameters were determined from individual animal data using non-compartmental analysis in WinNonlin (Version 5.2, Pharsight, CA). These included maximum observed concentration in plasma (C_max_), time to C_max_ (T_max_), area under the concentration-time curve from time zero to the last observed concentration (AUC_(0-t)_) and extrapolated to infinity (AUC_(0-∞)_), terminal half-life (t_1/2_), systemic clearance (CL), central distribution volume (V_c_), and volume of distribution at steady state (V_ss_). AUC values were calculated using the log-linear trapezoidal rule. CL was calculated as dose/AUC_(0-∞)_; terminal half-life was calculated as ln(2)/(slope of the terminal log-linear phase).

### Oral glucose tolerance test (OGTT) and weight loss studies

PF-05231023 was administered by either single IV or SC injection at 0.03, 0.1, 0.3, 1, 3, or 10 mg/kg to ob/ob mice. OGTTs were conducted in ob/ob mice on day 6 after dosing according to [19,20]. Briefly, mice were fasted overnight, and administered an oral gavage of 1 g kg^−1^ dextrose and blood glucose concentrations were measured using handheld glucometers at the indicated time points in figures. For weight loss studies, DIO mice were administered PF-05231023 IV using 1, 3, and 10 mg/kg once a week for three weeks and SC using 1 and 10 mg/kg of PF-05231023 once a week for three weeks. Body weight was measuredly twice per week throughout the study.

### PK/PD modeling for OGTT

PK modeling of PF-05231023 in ob/ob mice (schematically shown in S1a Fig.) was performed as follows. A single systemic compartment, representing 4% of the body weight of the animals (in agreement with measurements), was used for intravenous administration cases. For subcutaneous delivery a depot compartment was used, with first-order absorption characterized by an absorption half-life of 48 hours. The parent molecule and various metabolites were represented with an array of binary values [C_1_ N_1_ C_2_ N_2_], letters representing the appropriate terminus of FGF21, and subscript indicating which arm of the CovX-body includes that terminus. A 1 for a given value indicates that the appropriate terminus was intact, and a 0 indicates that it was clipped. For example, [1 1 0 1] represents a species with a fully intact FGF21 molecule (C and N terminus) on one arm, and a clipped C terminus/intact N terminus on the other arm. A dynamic simulation was performed where each intact terminus could independently be clipped, changing a 1 at that position to a 0; these reactions take place with first-order rate constants reflecting the measured half-life of the appropriate terminus, 2.5 and 13 hours for the C and N termini respectively; we assume these clipping processes occurred in depot and systemic compartments with identical half lives. Intact PF-05231023 and all metabolites were transported from the depot to the systemic compartment with the same rate constant above. An additional clearance corresponding to the observed half-life of the CovX-2000 backbone (~12 days) was applied to all molecules in the systemic compartment. For example, [1 1 0 1] in the systemic compartment participates in five reactions, one of which creates [1 1 0 1] from [1 1 1 1] with the C terminal half-life, three of which convert it into more degraded metabolites [0 1 0 1], [1 0 0 1], and [1 1 0 0], and the last of which clears the entire molecule from the systemic compartment:

$${d\left[1101\right]}_{systemic}/dt\;=\;\frac{\ln\left(2\right)}{48\;hr}{\left[1101\right]}_{depot}+\frac{\ln\left(2\right)}{2.5\;hr}{\left[1111\right]}_{systemic}-\frac{\ln\left(2\right)}{2.5\;hr}{\left[1101\right]}_{systemic}-\frac{ln(2)}{13\;hr}{\left[1101\right]}_{systemic}-\frac{ln(2)}{13\;hr}{\left[1101\right]}_{systemic}-\frac{ln(2)}{12\;days}{\left[1101\right]}_{systemic}$$

The first five terms have corresponding opposite fluxes with the species [1 1 0 1] generated in the depot and [1 1 1 1], [0 1 0 1], [1 0 0 1], and [1 1 0 0] in the systemic compartment respectively. This modeling scheme reproduced the time courses of total intact CT and total intact NT termini in the systemic compartment for both SC and IV administration, and further allowed calculation of the amount of intact FGF21 in a way not easily measurable experimentally, as a combined intact C and N terminal ELISA would identify (for example) [1 1 0 0] and [1 0 0 1] as containing both intact C and N termini while only the first species would actually contain a fully intact FGF21 molecule. The population of “intact” molecules was defined as the sum of all species containing at least one FGF21 molecule with both C and N termini unclipped. As internal hASC pERK data (not shown) suggests a similar response between one-armed and two-armed molecules, a fully intact PF-05231023 was only counted once. The population of “N-only” molecules was defined as the sum of all molecules not containing any intact FGF21 but still bearing at least one intact N terminus.

Considering the relatively stable OGTT response between days 3 and 4 post dosing, while the levels of PF-05231023 and its metabolites are dropping, a pharmacodynamics model architecture where the OGTT effect is determined by the convolution of instantaneous signaling (characterized by a simple hyperbolic efficacy expression) with an exponential decay was selected. The instantaneous signaling S(t) by both the intact and N-only molecule pools is given by

$$S\left(t\right)\;=\;\frac{\frac{\left[I\right]}{K_{I}}+\frac{\left[N\right]}{K_{N}}}{1+\frac{\left[I\right]}{K_{I}}+\frac{\left[N\right]}{K_{N}}}$$

with [*I*], [*N*], *K*_*I*_, and *K*_*N*_ representing the concentrations of intact and N-only molecule pools and the effective half-maximal response concentrations for intact and N-only pools respectively. *K*_*I*_ was set to 0.5 nM, reflecting the *in vitro* pERK response EC50 in hASC cells and the value of *K*_*N*_ was subject to optimization. The OGTT response was determined by the convolution

$$OGTT\left(t\right)\;=\;A\int_{0}^{t}S\left(\tau \right)e^{-\left(t-\tau \right)/\gamma }d\tau$$

The time constant *tau* representing the decay of the signaling effect persistence was optimized. The normalization *A* was set by matching the model output at a selected time to the experimental data; this factor is then fixed for the remaining time points.

The OGTT effect was defined by the difference in AUC between treatment and control for plasma glucose between time 0 and 120 minutes following glucose challenge. Optimization of the parameters *K*_*N*_ and *γ* was performed by minimizing the objective function defined by the summed squared differences between the data and model, normalized to data.

### PK/PD modeling for BW loss in DIO mouse

Body weight modeling in DIO mice was performed in two stages. First, a pharmacokinetic model (S1b Fig.) was constructed to generate time courses of intact and N-only molecules in adipose (the assumed target tissue). This model consists of two compartments: a peripheral compartment representing the extracellular space in adipose tissue with volume 0.8 ml (assuming 20 g of fat with 4% extracellular space), and a central compartment with volume 2.4 ml (based on a 45 g body weight and 7% total V_ss_ as determined experimentally by the intact NT assay). A first-order transport rate of 0.0167 ml/hr was symmetrically applied between these compartments. This rate corresponds to the fat extracellular volume exchanging with the plasma over 48 hours. For IV administration, the full dose was delivered to the central compartment. For subcutaneous administration, the dose was instantaneously added to a depot which transfers to the central compartment with a characteristic time (i.e., half-life) of 48 hours. In all compartments (central, peripheral, and depot for subcutaneous delivery), the DIO mouse-measured half-lives of 1.7 hours and 26.2 hours were assumed for the intact CT and NT respectively. Following intact CT cleavage, an “N-only” metabolite was generated; it was assumed that N-terminal cleavage resulted in a completely inactive molecule, see for example [15]. The clearance of the antibody “backbone” was ignored as it was anticipated to be much longer than the intact NT stability (12 days for the backbone vs 13 hours for the intact NT in ob/ob mice). In contrast to the ob/ob PK modeling above, this simplified approach was utilized owing to the larger difference between intact CT and NT half-lives (meaning intact C/cleaved N molecules could reasonably be neglected). This PK model leads to calculated bioavailabilities of 2.6% and 38.5% for intact CT and NT respectively following subcutaneous dosing, a T_max_ ~24 hours for the intact NT following SC delivery, and a buildup in fat-to-plasma exposure over 5 days resulting in maximal ratio after around 7 days, all in agreement with experimental observations (fat-to-plasma ratio following single SC or IV dosing, data not shown). In contrast to the PK modeling for the ob/ob mice, the outputs of this PK model were time courses in extracellular adipose of intact and N-only PF-05231023 molecules.

PD modeling for body weight consisted of several steps. First, given the PK previously determined, receptor occupancy was calculated in the adipose. The extracellular receptor complex (nominally consisting of KLB and FGFR1c) concentration was set at 4.15 nM; this corresponds to 10,000 sites per adipocyte, with 100 pl cell volume and assuming 4% extracellular space (i.e., 4 pl/cell). The affinity of PF-05231023 for this complex was set to 0.5 nM based on *in vitro* hASC measurements. As the precise nature of the N-only metabolite pool was not known, its effective affinity was estimated by the model by optimization for best fit. It was assumed that within the fat space, rapid equilibrium was achieved between intact, N-only, and receptor, by numerically solving the following system of equations for each dose and time:

$$\begin{array}{l} {\left[I\right]}_{free}\cdot {\left[R\right]}_{free}=K_{D,I}\cdot \left[IR\right]\\ {\left[N\right]}_{free}\cdot {\left[R\right]}_{free}=K_{D,N}\cdot \left[NR\right]\\ {\left[I\right]}_{total}={\left[I\right]}_{free}+\left[IR\right]\\ {\left[N\right]}_{total}={\left[N\right]}_{free}+\left[NR\right]\\ {\left[R\right]}_{total}={\left[R\right]}_{free}+\left[IR\right]+\left[NR\right] \end{array}$$

Receptor occupancy (*RO*) was calculated as ([*IR*]+[*NR*])/[*R*]_*total*_. Both intact and N-only were assumed to have equal signaling potential (identical E_max_ values) as E_max_ has been shown *in vitro* to depend on N-terminal status rather than C-terminal [15].

A sigmoidal transfer function was used to relate receptor occupancy to an instantaneous downstream effect *P*(*t*) (with parameters *ρ* and *δ* to be optimized later, and normalization *A* giving *P*(*t*) = 1 at *RO*(*t*) = 1):

$$P\left(t\right)\;=\;\frac{A∙{\left(RO(t)\right)}^{\rho }}{\delta^{\rho }+{\left(RO(t)\right)}^{\rho }}$$

Body weight gain or loss was determined by the difference between calories consumed and calories burned. Internal data (not shown,) shows that total adipose tissue (TAT) is related to body mass over the range of body weights in this study by the relationship

$$TAT\left(g\right)\;=\;0.46∙BW\left(g\right)-9.9g$$

This finding is consistent with published data [21]. Body fat contains approximately 39.5 kJ/g, and lean tissue contains 7.6 kJ/g [22], therefore, the net caloric balance in one of these mice can be expressed as

$$∆E\;=\;∆BW\left(0.46∙39.5\frac{kJ}{g}+0.54∙7.6\frac{kJ}{g}\right)$$

The resting metabolic rate (RMR) in mice under a range of body weights and dietary conditions has been measured as 0.86 kJ/(g fat free mass) [23]. Fat free mass can be determined by the difference between BW and TAT. Food intake (FI, in kcal/day) for the control groups of mice in this study was estimated by smoothing the body weight data (to reduce the effect of fasting prior to alternate measurements for glucose determinations), and adding the resting metabolic rate to the energy content of added tissue:

$${FI}_{control}\left(t\right)\;=\;RMR∙FFM\left(t\right)+\frac{dBW}{dt}\left(0.46∙7.6\frac{kJ}{g}+0.54∙39.5\frac{kJ}{g}\right)$$

Prior work (Tristan Maurer & Dennis Scott, unpublished results) has suggested that following a weight loss regimen, an overfeeding response exists which attempts to restore body weight to its nominal value (in this case, the vehicle group body weight); this overfeeding response can be characterized by

$${FI}_{treatment}\left(t\right)\;=\;{FI}_{control}(t)∙{\left(\frac{{BW}_{control}}{{BW}_{treatment}}\right)}^{\beta }$$

Precise measurements of food intake were not able to be obtained in this study; however, it generally appeared that treated animals consumed approximately the same amount of food per day throughout the treatment period as at baseline. To combine the observation with a mechanism for body weight rebound during washout, the following equation was used to determine food intake in the treated animals:

$$FI\left(t\right)\;=\;FI\left(t\;=\;0\right)∙P\left(t\right)+{FI}_{control}∙{\left(\frac{{BW}_{control}}{{BW}_{treatment}}\right)}^{\beta }∙(1-P\left(t\right))$$

Here, *P*(*t*) is the efficacy as a function of receptor occupancy described above, and *beta* is a parameter to be determined. Under maximum signaling (*P* = 1), the food intake matches the baseline intake at t = 0; in the absence of signaling, the full overfeeding response is observed until the treatment group matches the control group at which time both groups will have identical food intake.

A proposed mechanism of action for FGF21 in rodents is “browning” of white adipose, resulting in energy utilization via thermogenesis [24]. Therefore, in this model, an additional energy consumption term is added:

$${∆E}_{FGF21}(t)\;=\;-\varepsilon ∙P(t)∙TAT(t)$$

*ε* is an “E_max_”-like parameter to be determined, and *P*(*t*) is the same efficacy function as above. The effect is assumed to be proportional to the amount of body fat. The total energy balance then becomes

$$∆E\left(t\right)\;=\;FI\left(t\right)-RMR∙FFM\left(t\right)-{∆E}_{FGF21}(t)$$

Given the body composition determined above, the change in body weight is then calculated as

$$\frac{dBW}{dt}\;=\;\frac{∆E(t)}{0.46∙39.5\frac{kJ}{g}+0.54∙7.6\frac{kJ}{g}}$$

## Results

### PK properties of PF-05231023

**Fig 1:**
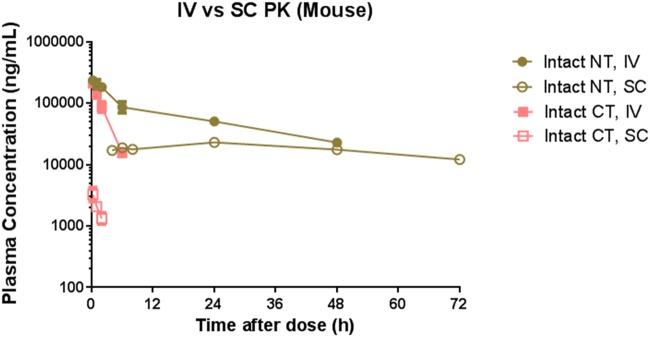
Plasma concentration-time curves of PF-05231023 intact CT and NT. Plasma **concentration-time curves** of PF-05231023 in DIO mice following a single IV and SC administration at 10 mg/kg. 3 animals were used per time point. n = 3 animals were used for the 10 mg/kg IV and n = 4 animals were used for the rest of the groups.

The mean IV and SC PK of the intact CT and NT are presented in Table 1 and Table 2, respectively. The mean concentration-time profiles in DIO mice are shown in Fig. 1. With the intact NT assay, systemic CL of PF-05231023 in mice was 2.62–3.25 ml/h/kg, V_c_ was 22.5–40.8 ml/kg, V_ss_ was 40.6–70.5 ml/kg, the terminal elimination half-life (t_1/2_) was 13.0–22.7 hours after a single IV dose. After SC administration, the terminal t_1/2_ was estimated to be 55.1 hours in DIO mice (not calculated for ob/ob mice due to insufficient data points); C_max_ was about 10x fold less than those observed after IV administration; T_max_ was observed at 19.0–21.0 hr; and SC bioavailability was estimated to be 44.2–58.6%. In contrast, with the intact CT assay, we observed ~10x higher systemic CL (18.8–20.2 ml/h/kg), similar V_c_ (24.2–42.2 ml/kg), whereas ~2x lower V_ss_ (28.1–38.1 ml/kg), and > 10x shorter t_1/2_ (1.6–2.2 hours) following a single IV dose. After SC administration, T_max_ of the intact CT was observed 4–6 hr post dose; t_1/2_ was 2.48–3.61 hr; SC bioavailability was 4.14–7.70%, which is about 6–10x lower than that estimated for the intact NT. The observed C_max_ of the intact CT following SC administration was 60 to 70 times lower than those observed following IV administration.

**Table 1:** Summary of key PK Parameters of PF-05231023 in DIO mice following IV administration.

Assays	Mouse strain	Cl (ml/hr/kg)	V_c_ (ml/kg)	V_ss_ (ml/kg)	t_1/2_ (h)	C_max_ (μg/ml)	AUC_(0-t)_ (μg•h/ml)	AUC_(0-∞)_ (μg•h/ml)
Intact CT	ob/ob	20.2 ± 3.66	24.2 ± 6.45	28.1 ± 4.45	2.20 ± 0.99	339 ± 76.4	438 ± 78.1	448 ± 72.2
	DIO	18.8 ± 2.7	42.2 ± 6.19	38.1 ± 9.37	1.61 ± 0.20	209 ± 32.9	502 ± 84.3	538 ± 77.1
Intact NT	ob/ob	3.25 ± 1.72	22.5 ± 5.66	40.6 ± 8.23	13.0 ± 10.3	390 ± 86.4	1730 ± 664	3640 ± 2550
	DIO	2.62 ± 0.12	40.8 ± 3.81	73.2 ± 9.29	22.7 ± 3.64	235 ± 26	3060 ± 176	3820 ± 165

CL = Total plasma clearance; V_c_ = Central distribution volume; V_ss_ = Volume of distribution at steady state; t_1/2_ = elimination half-life; T_max_ = time to reach C_max_; C_max_ = Maximum observed serum concentration; AUC_(0-t)_ = Area under concentration-time curve from 0 to last measured time point; AUC_(0-∞)_ = area under concentration-time curve from 0 to infinity postdose. Values presented are mean ± STDEV with n = 3 for 10 mg/kg IV and n = 4 for the rest of the groups.

**Table 2:** Summary of key PK Parameters of PF-05231023 in DIO mice following SC administration.

Assays	Dose (mg/kg)	t_1/2_ (h)	T_max_ (h)	C_max_ (μg/ml)	AUC_(0-t)_ (μg•h/ml)	AUC_(0-∞)_ (μg•h/ml)	F (%)
Intact CT	ob/ob	2.48 ± 0.70	6.00 ± 0.00	4.53 ± 0.84	32.7 ± 9.89	34.5 ± 9.06	7.70
	DIO	3.61 ± 1.36	4.00 ± 0	3.26 ± 0.98	15.3 ± 3.35	22.3 ± 3.08	4.14
Intact NT	ob/ob	NC	21.0 ± 6.00	44.7 ± 1.74	765 ± 107	NC	44.2^a^
	DIO	55.1 ± 12.8	19.0 ± 10.0	24.1 ± 2.13	1280 ± 158	2240 ± 288	58.6

t_1/2_ = elimination half-life; T_max_ = time to reach C_max_; C_max_ = Maximum observed serum concentration; AUC_(0-t)_ = Area under concentration-time curve from 0 to last measured time point; AUC_(0-∞)_ = area under concentration-time curve from 0 to infinity postdose; F = bioavailability, was calculated based on both AUC_(0-t)_ and AUC_(0-∞)_ and both values were reported (mean sc AUC_(0-t)_ /mean IV AUC_(0-t)_) % / (mean SC AUC_(0-∞)_ /mean iv AUC_(0-∞)_) %. Values presented are mean ± STDEV with n = 3 for 10 mg/kg IV and n = 4 for the rest of the groups. NC: Not calculated due to insufficient data.

### PF-05231023 causes improvement of glucose excursion in a glucose tolerance test

**Fig 2:**
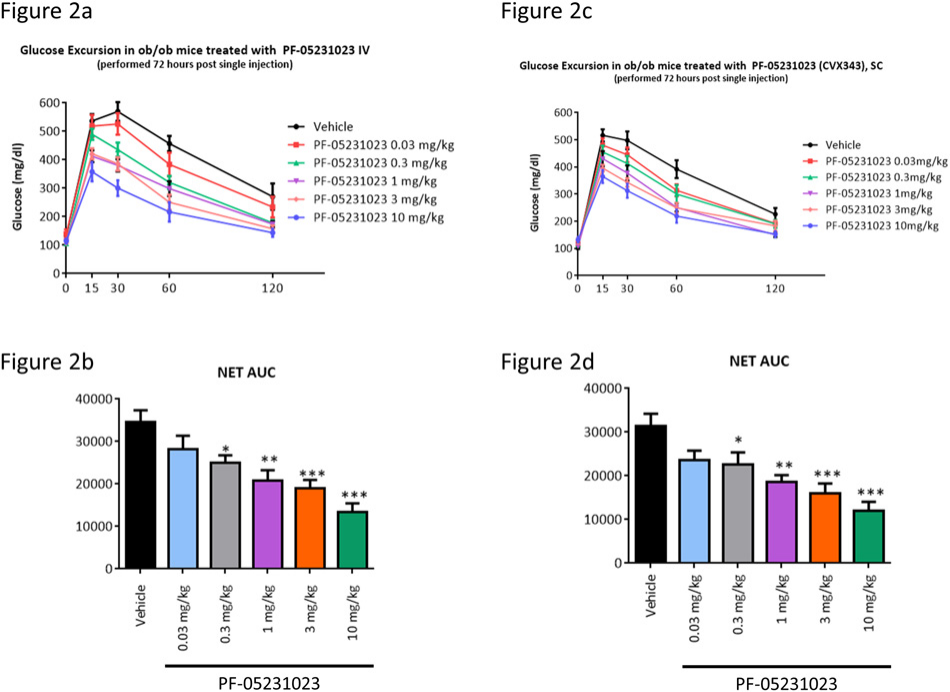
Glucose lowering during OGTT in ob/ob mice upon IV and SC administration of PF-05231023. Male ob/ob mice approximately 9 weeks of age were administered PF-05231023 IV (a) or SC (c) and OGTT was performed 72 hours later, after an overnight fast. At least 8 mice were used per dose group for the OGTTs. Area under the curve during OGTT after IV (b) or SC (d) administration of PF-05231023. 8 animals were used per group. * p < 0.05, ** p < 0.01, and *** p < 0.001 using one-way Anova with Dunnett’s post hoc test.

We performed an OGTT in ob/ob mice after a single IV (Fig. 2a) or SC (Fig. 2c) administration of PF-05231023 at indicated doses. PF-05231023 administered at 0.03 mg/kg displayed a trend towards decreased AUC which did not achieve significance compared to vehicle. All other doses administered either IV (Fig. 2b) or SC (Fig. 2d) caused a dose-dependent and significant decrease in glucose AUC compared to control. Moreover, despite low SC bioavailability of intact FGF21 CT, effects of PF-05231023 in glucose lowering during OGTT lowering were similar between IV and SC administration for the entire dose range tested. These results demonstrate PF-05231023 is equally efficacious in improving glucose tolerance in ob/ob mice when administered either IV or SC.

**Fig 3:**
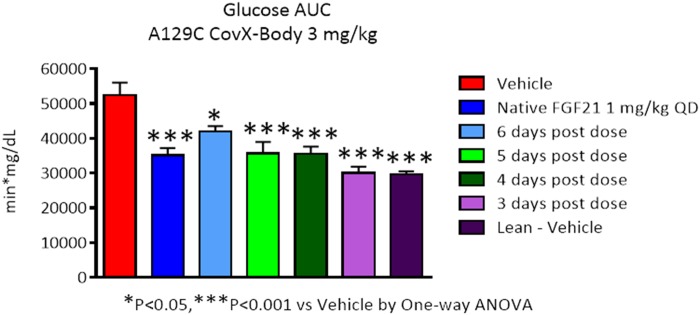
Glucose lowering during OGTT after PF-05231023 administration. Male ob/ob mice approximately 9 weeks of age were administered 3 mg/kg PF-05231023 SC and OGTT was conducted after an overnight fast on the indicated days. At least 8 animals were used per group per day. * p < 0.05, ** p < 0.01, and *** p < 0.001 using one-way Anova with Dunnett’s post hoc test.

Next, we determined the durability of the glucose lowering effect of PF-05231023 in ob/ob mice upon administration of a single 3 mg/kg SC dose by performing OGTTs from day 3 to day 6 post-dose. As expected, lean animals administered vehicle had a significantly lower glucose AUC during OGTT, compared to obese animals administered vehicle (Fig. 3). PF-05231023 caused a significant decrease of glucose AUC during OGGT 3 days after dosings that was comparable to lean animals. On days 4, 5 and 6 PF-05231023 caused a significant reduction in AUC compared to obese animals administered vehicle. However, the decrease of AUC appeared to taper off on these days, compared to day 3, although the numbers did not achieve significance. On day 6, decrease of AUC was significantly lower than obese animals administered vehicle, but trended higher than all other treated groups and lean control. These results demonstrate that a single SC dose of PF-05231023 causes a robust decrease in glucose AUC 3 days after dosing that is comparable to lean animals.

### PF-05231023 causes weight loss in DIO mice

**Fig 4:**
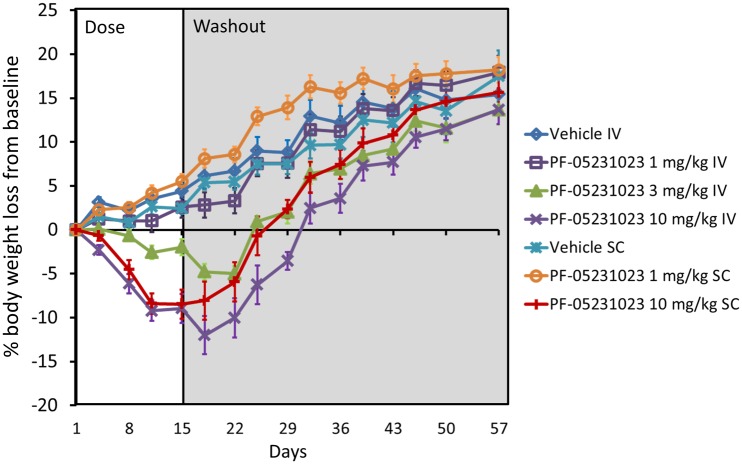
Percentage BW loss in DIO mice following IV and SC administration of PF-05231023. Male DIO mice at approximately 14 weeks on 60% HFD were administered PF-05231023 IV or SC at the indicated doses on Day 1, 8 and 15. Body weight was measured twice a week throughout the course of the study until day 57. At least 9 animals were used per dose group.

DIO mice were treated IV using 1, 3 and 10 mg/kg PF-05231023 once a week for three weeks and SC using 1 and 10 mg/kg of PF-05231023 once a week for three weeks Fig. 4. Body weight was measured twice per week throughout the study. 1 mg/kg administered SC and IV had no effect on BW compared to control. 3 mg/kg IV caused modest weight loss compared to control which achieved nadir on day 22, after which BW rebounded crossing baseline approximately on Day 22. 10 mg/kg SC and IV caused similar and robust weight loss until Day 15, after which the SC dosed group started rebounding and crossed baseline on Day 25. 10 mg/kg dosed IV lost an additional amount of weight until Day 18, after which the animals started rebounding and crossed baseline on Day 32. At the end of study, all groups that lost weight gained back similar body weight compared to control animals administered vehicle, regardless of the route of administration. These data demonstrate that PF-05231023 causes robust weight loss when administered once a week for three weeks.

### PK/PD modeling to integrate the OGTT and BW loss observed in mice

**Fig 5:**
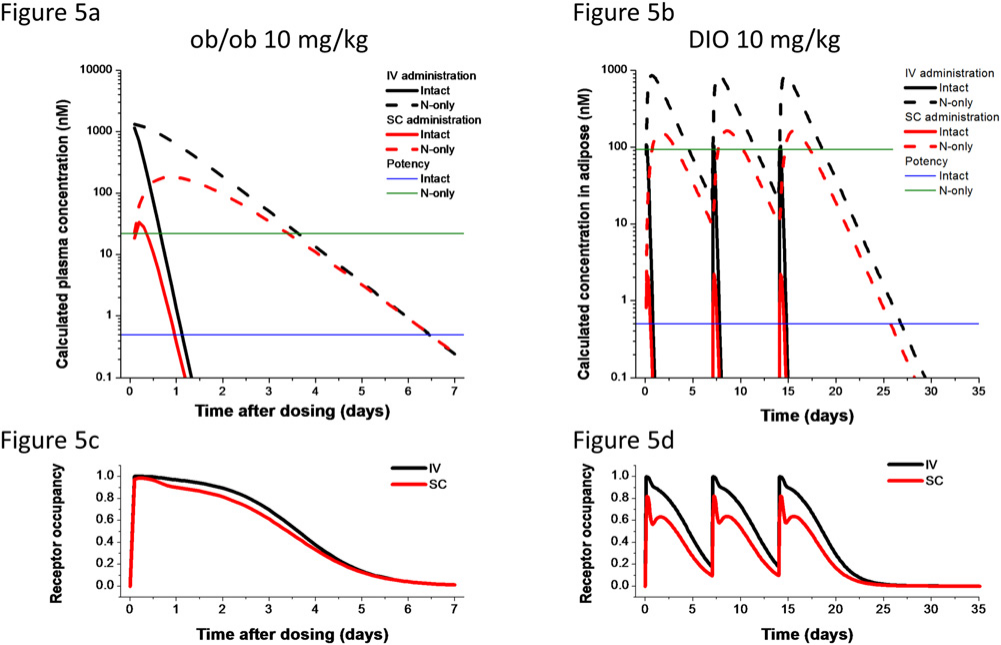
PK and RO time courses for modeled scenarios. (a,b) Calculated intact (solid line) and N-only (dashed line) following IV (black) or SC (red) administration of 10 mg/kg of PF-05231023. The *in vitro* potency of the intact molecule is shown in blue, and the estimated *in vivo* potency of the N-only pool is shown in green. As the model includes linear PK, other doses can be considered by simple shifting of the curves up or down by the relative dose. The results for central compartment levels in the ob/ob mouse model are in (a); the curves for adipose exposure in DIO mice are in (b). (c,d) Calculated receptor occupancy in the central space of ob/ob mice (c) or the adipose space of DIO mice (d) for the 10 mg/kg dose delivered IV (black) or SC (red). The rapid but short-lasting rise in RO following dosing driven by intact molecules is evident, followed by a sustained RO by the N-only molecules.

The data set used for OGTT model optimization was the response to a single 3 or 10 mg/kg SC dose on days 3, 4, 5, and 6 post-dose. The PK model described above was used to estimate the size of the intact and N-only pools. These predictions are shown for 10 mg/kg doses delivered either SC or IV in Fig. 5a, with calculated receptor occupancies in Fig. 5c. The calculations for BW in DIO is shown in Fig. 5b, with calculated receptor occupancy shown in Fig. 5d. The normalization *A* was set by matching the day 3 response to the 10 mg/kg dose. *K*_*N*_ was optimized at 22 nM (compared to 0.5 nM for *K*_*I*_) and *γ* was optimized at 3 days.

**Fig 6:**
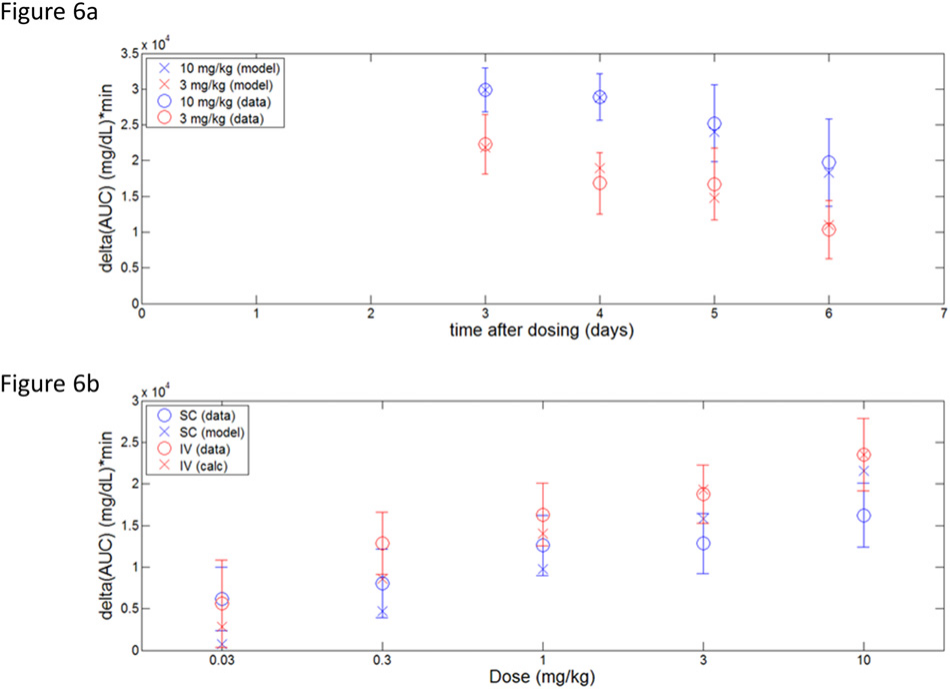
OGTT modeling in ob/ob mice. (a) Data and model predictions for OGTT performed 3, 4, 5, and 6 days following single 3 (red) or 10 (blue) mg/kg SC doses of PF-05231023. (b) Day 3 dose response to single 0.03–10 mg/kg PF-05231023 delivered IV (red) or SC (blue) on day 0. Data represented by circles and error bars; model predictions represented by x’s.

The optimized model predictions are represented in Fig. 6a. For further validation, a dose response to IV- or SC-administered PF-05231023 on day 3 was simulated and compared to data in Fig. 2 and comparison in Fig. 6b. In this case the model output normalization was set to the 10 mg/kg IV dose. Comparing the calculated exposures to the estimated potency values for intact and N-only species, it is clear that while the intact molecules may contribute to a fast onset of effect following dosing, the majority of OGTT effect at longer times is driven by the N-only pool.

**Fig 7:**
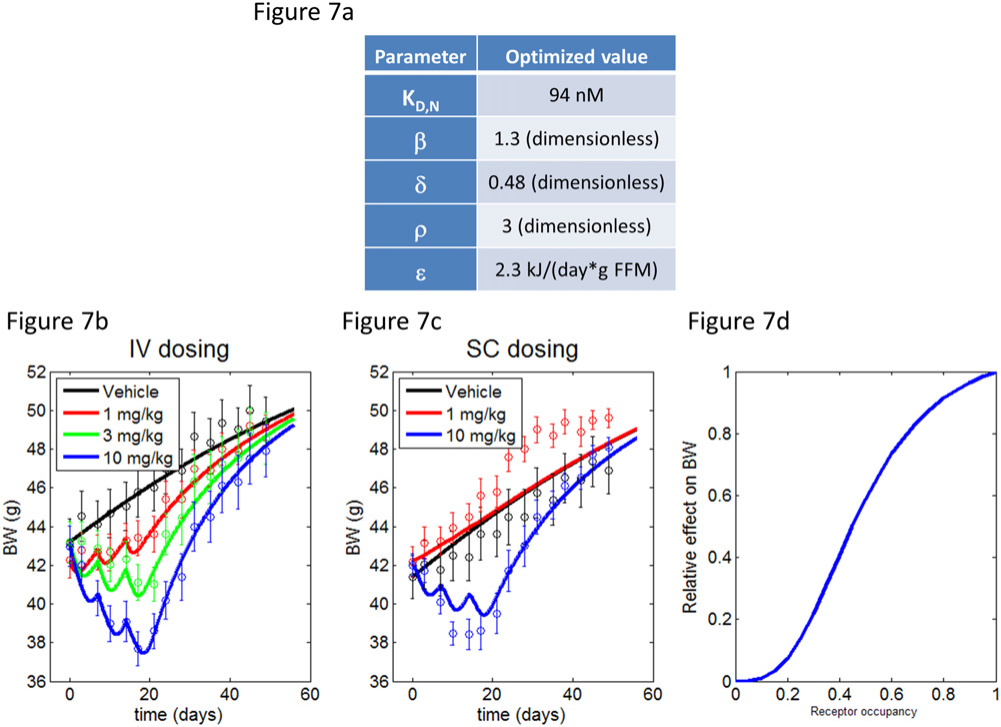
BW loss model results in DIO mice. (a) Optimized model parameter values, as described in the text. (b,c) Data (shapes) and model predictions (curves) for BW changes following three once-weekly doses of PF-05231023 delivered IV (b) or SC (c) starting on day 0. (d) Optimized transfer function (function of *ρ* and *δ*; P(RO) in the text) relating the effect on BW changes to receptor occupancy.

The body weight model was simultaneously fit to all five treatment conditions (1, 3, 10 mg/kg QW IV and 1, 10 mg/kg QW SC), the vehicle data from which had already been used to determine *FI*_*control*_. An objective function was defined as the sum of squared differences between model output and treatment data, normalized to data. The parameters *K*_*D*,*N*_, *beta*, *δ*, *ρ* and *ε* were optimized to minimize this objective function. The final fit values are given in Fig. 7a and the optimized model predictions are shown in Fig. 7b and Fig. 7c. The estimated right shift in potency compares reasonably with the *in vitro* right shift of a single C terminal truncated FGF21 molecule (2–171). The function *P*(*RO*) is displayed in Fig. 7d.

## Discussion

FGF21 has generated interest as a potential therapeutic against metabolic disease. Administration of FGF21 causes metabolic improvement in all species in which it has been reported so far, including humans [3,6,1,18]. Given the short half-life of native FGF21 limiting its therapeutic utility for chronic indications, various half-life extending technologies have been employed to generate long-acting FGF21 [18]. Renal filtration is considered to be the major clearance mechanism for administered recombinant FGF21 (rFGF21) and endogenous FGF21 in man [25,26]. Therefore, prevention of renal filtration has been adopted as an approach for half-life extension by various groups, including PEGylation [17], Fc-fusion [18] and conjugation to CovX-2000 [6]. As expected, all reported FGF21-conjugates have shown prolonged *in vivo* half-life compared to native FGF21, demonstrated by using PK assays that are unable to differentiate between intact FGF21 molecule and protease-clipped metabolites [13,6].

We generated a novel long acting FGF21 analog termed PF-05231023, which is a complex molecule developed by covalent conjugation of two modified recombinant human (rh)FGF21 to a non-targeting antibody scaffold, CovX-2000 body (CovX body) as described previously [6]. The rFGF21 is conjugated to CovX body at the mid-region of the FGF21 protein, at A129C. This conjugation site was shown to have minimal impact on the in vitro potency [8], consistent with the known binding motifs to KLB and FGFR1c were at the C- and N-terminus, respectively, thus both distal from the conjugation site. This molecule is such that in each molecule of PF-05231023, there are two free FGF21 C- and N-termini respectively. The pharmacological effects of FGF21 are mediated through FGFR1c/KLB [9,5] and both intact CT and NT are required for formation of FGF21/KLB/FGFR1C complex [27].

To better understand PK/PD relationship of PF-05231023, we developed two assays to independently measure the circulating levels of intact NT and CT, respectively. The Cl and t_1/2_ of the intact CT and NT are much faster compared to those determined using an ELISA that had specificity for the mid-region of FGF21 [6], suggesting that conjugation of FGF21 to CovX body has shifted the primary Cl mechanism from renal filtration to protease-mediated degradation (Giragossian et al., Pfizer, submitted manuscript). The circulation levels of intact CT is undetectable starting at 6–12 h post either IV or SC dose; whereas circulation levels of intact NT is present up to 48–72 hr post dose, suggesting that the major circulating species are metabolites with cleaved CT starting from 6~12 hr post either SC or IV dose. Interestingly, effects of PF-05231023 on glucose lowering during OGTT and BW loss in DIO mice were similar following administration of PF-05231023 at the same dose level administered either IV or SC. A similar PD response between IV and SC administration would only be expected for molecules with reasonable SC bioavailability (e.g. ≥ 50%). According to PK data, the SC bioavailability for the intact NT was ~44–59%, whereas the SC bioavailability of intact CT was only 4–7.7%; indicating circulation levels of intact CT is significantly lower following SC, compared to IV administration. The observation of similar glucose lowering effect during OGTT between IV and SC administration of PF-05231023 across a wide dose range between 0.3 to 10 mg/kg, could only be explained by hypothesizing that the intact NT related drug species, rather than the intact CT, are the key driver(s) for the observed effects. To test this hypothesis, PK models with integration of PK properties of both intact CT and NT was developed, where each PF-05231023 molecule contains two intact CT and two intact NT. Therefore, an ELISA with anti-intact CT as capture antibody and anti-intact NT as detection antibody, or vice versa, would not be able to differentiate between true intact FGF21 molecules (fully functional) with intact NT and CT on the same arm (e.g. [1111], [1011], [1101]) from pseudo-intact (or functionally impaired) molecules with intact CT and NT on separate arms (e.g. [1001] and [0110]). Our strategy of independent measurement of PK of the intact NT and CT separately allows the utilization of sophisticated PK modeling to calculate all potential circulating species that could still be active as shown in the schematic in S1 Fig. The rate limiting cleavage site of FGF21 was identified in the C-terminus of FGF21 between Pro171 and Ser172 [13], that should render the protein from being detected in the intact CT assay, but still be detected in the intact NT assay. According to both internal and external ERK-phosphorylation assay data, truncated FGF21 with an intact NT still retains full activity albeit with reduced potency [14]. Based on the above, we generated the PK model to allow for estimation of total circulation levels of two pools of bioactive PF-05231023 species, intact, which is expected to act with high potency and N-only, which is expected to act with an impaired potency.

As discussed, PF-05231023 is a long-acting FGF21 analog which is unique in that the molecule results in in vivo generation of an intact N and C terminal end that allows us to use this molecule as a tool compound to begin to explore the biology and specific contributions of each of the termini to its pharmacologic actions. The mechanisms by which FGF21 confers weight loss and glycemic control, appear to be mediated through different tissues. Adipose tissue plays an important role in FGF21 pharmacology since both FGFR1 and KLB are expressed in these cells [9,5,28] and lipodystorphic mice are refractory to the beneficial effects of FGF21 administration [29]. Further evidence of the adipose playing an important role in mediating actions of FGF21 is demonstrated by the fact that adipocyte-specific deletion of FGFR1 or KLB ameliorates the metabolic effects of pharmacological FGF21 administration [5,30]. Moreover, studies have shown that adiponectin is required for glycemic control and insulin sensitizing effects of pharmacological administration of FGF21 in obese mice suggesting adipose tissue as the primary driver for glycemic control [31,32]. Consistent with these findings, FGF21 administration in humans increased in adiponectin [2], although this could be in part, secondary to weight loss observed in the subjects. In this study, the effect of FGF21 on glucose lowering was assumed to be more relevant to systemic drug exposure. The calculated exposure of intact vs. N-only was shown in Fig. 6A.

The mechanism by which FGF21 causes weight loss is more complex. In mice [8] and rats [33], FGF21 administration causes increased food intake when expressed per unit body weight suggesting a hyperphagic response, implicating peripheral energy expenditure as a central mechanism by which FGF21 causes weight loss in these species [33,8]. In non-human primates, an FGF21 analog causes a marked reduction in food intake [34], although a monoclonal antibody against KLB/FGFR1c does not appear to cause significant changes in food intake, although imparting robust weight loss [5]. In addition, leptin appears to play an important role in mediating weight loss upon FGF21 administration, since ob/ob and db/db mice have little to no weight loss upon FGF21 administration [35,6,36,37]. Moreover, a recent report demonstrates that SCN KLB KO mice administered FGF21 do not lose BW, clearly demonstrating the direct contribution of the CNS in mediating weight loss upon FGF21 administration [38]. Although Lilly reported weight loss in humans [2], food intake was not measured in the study. Further clinical studies are required to determine the impact of FGF21 administration on food intake and help align the species differences reported, to help understand the mechanism.

Aspects of model structure and optimized parameters for OGTT and BW modeling provide a basis for hypothesis generation regarding the underlying mechanisms for glucose and weight regulation. The modeled compartment for action for body weight in the DIO mouse model is the fat, consistent with the putative mechanism for energy expenditure and beiging of white adipose [24]. Interestingly, the modeled effect on food intake also depends on exposure in this tissue and in fact is driven by the same concentration-effect relationship characterized by the same parameters, suggesting that suppressed food intake, generally speculated to be CNS-driven, may arise because of a messenger arising in the fat. By comparison, the OGTT effect is better described when assuming effects driven by the central compartment exposure, suggesting a different mechanism based on the different site of action. The different relative potency of the N-terminal metabolite pool also suggests that the molecular target for PF-05231023 action is different between glucose and body weight regulation, although the difference may also represent different composition of the N-only pools between DIO and ob/ob mice.

The combined results of the PK/PD modeling also offer some insights into design of potential FGF21-based therapeutics. First, in both model systems, C-terminal clipped (N-only) FGF21 molecules were able to drive beneficial effects, although with substantially (approximately 40 to 200 fold) reduced potencies compared to intact FGF21 molecules. In these studies, the extension of the half-life of these N-only molecules was sufficient to sustain exposures in the efficacious range, enabling pharmacological effects over extended times, which is a desirable feature for injected therapeutics. The short lived but more potent intact molecules may lead to a rapid initiation of PD effects. An ideal FGF21 therapeutic would combine the advantages of the potent intact species with the long-lived N-only species, perhaps by stabilizing FGF21 against C-terminal proteolytic cleavage while preserving the protection against renal filtration enabling the long N-terminal half-life seen with PF-05231023. Such a molecule would possess immediate and extended pharmacology, allowing more favorable dosing paradigms such as reduced amount or frequency of administration.

## Supporting Information

S1 Fig Schematic for PK. (a) PF-05231023 processing in ob/ob mice. The C and N termini of each FGF21 molecule attached to the CovX scaffold are independently and sequentially processed, resulting in a mixture of various FGF21 species. Molecules containing an entire intact FGF21 are on a green background and are summed to give the “intact” concentration, and molecules without an intact FGF21 but at least one remaning N terminus are on a blue background and combine to give the “N-only” pool. The numerical labels for each species are as described in the text. Although not explicitly shown, the network of reactions here occur in the SC injection space as well as in the central volume, with first order absorption (same t_abs_) of all species from the injection space to the central. (b) PK model in DIO mice. In the SC depot, central, and extracellular adipose space, C-terminal clipping can take place converting from intact to N-only. Both forms can be N-terminal processed leading to effective clearance of the active forms. Time scales for absorption from SC space (t_abs_) and equilibration between central and adipose spaces (t_fat_) are shown. Within the adipose, the intact and N-only forms can bind to the receptor complex with indicated binding affinities.
(TIF)
