# Supplementary figures and images for: Pharmacokinetics (PK), Pharmacodynamics (PD) and Integrated PK/PD Modeling of a Novel Long Acting FGF21 Clinical Candidate PF-05231023 in Diet-Induced Obese and Leptin-Deficient Obese Mice
Source: PLoS One. 2015 Mar 19;10(3):e0119104. doi: 10.1371/journal.pone.0119104 (PMC4366384; doi:10.1371/journal.pone.0119104)

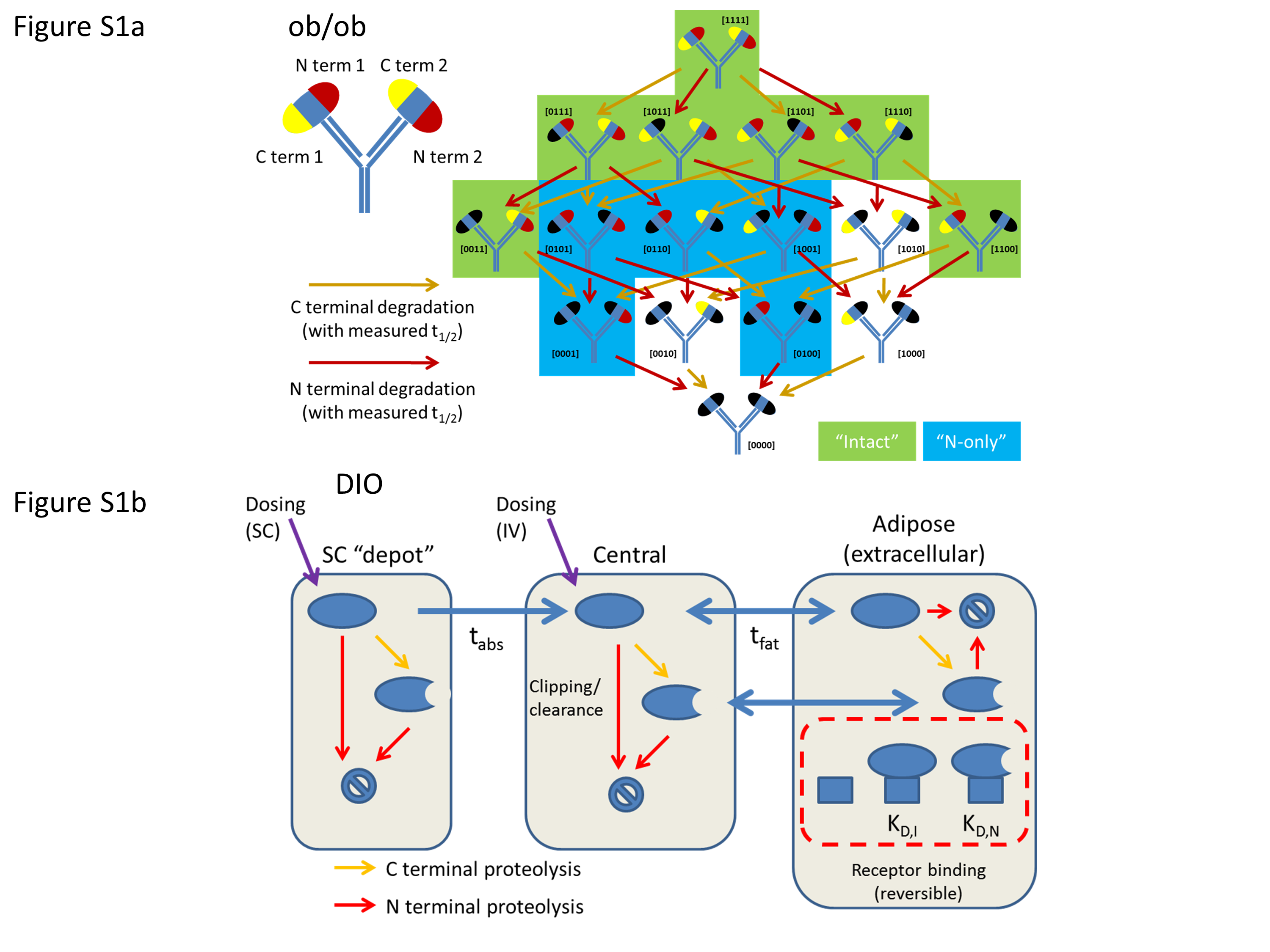

Supplement: S1 Fig — (a) PF-05231023 processing in ob/ob mice. The C and N termini of each FGF21 molecule attached to the CovX scaffold are independently and sequentially processed, resulting in a mixture of various FGF21 species. Molecules containing an entire intact FGF21 are on a green background and are summed to give the “intact” concentration, and molecules without an intact FGF21 but at least one remaning N terminus are on a blue background and combine to give the “N-only” pool. The numerical labels for each species are as described in the text. Although not explicitly shown, the network of reactions here occur in the SC injection space as well as in the central volume, with first order absorption (same tabs) of all species from the injection space to the central. (b) PK model in DIO mice. In the SC depot, central, and extracellular adipose space, C-terminal clipping can take place converting from intact to N-only. Both forms can be N-terminal processed leading to effective clearance of the active forms. Time scales for absorption from SC space (tabs) and equilibration between central and adipose spaces (tfat) are shown. Within the adipose, the intact and N-only forms can bind to the receptor complex with indicated binding affinities. (TIF) [file pone.0119104.s001.tif]
